# Predictive Risk Factors of Intestinal Necrosis in Patients with Mesenteric Venous Thrombosis: Retrospective Study from a Single Center

**DOI:** 10.1155/2019/8906803

**Published:** 2019-05-07

**Authors:** Yong Wang, Rui Zhao, Lin Xia, Ya-Ping Cui, Yong Zhou, Xiao-Ting Wu

**Affiliations:** Department of Gastrointestinal Surgery, West China Hospital, Sichuan University, Chengdu, China

## Abstract

**Purposes:**

Mesenteric venous thrombosis (MVT) is a serious condition. The current study aimed to identify risk factors of intestinal necrosis (IN) in patients with MVT to predict the onset of patients.

**Methods:**

Data pertaining to patients diagnosed with MVT between 2014 and May 2018 were reviewed. Patients' characteristics and risk factors of IN were assessed.

**Results:**

Seventy-eight patients were included in our study, of whom all cases were diagnosed as superior mesenteric venous thrombosis. There were fifty-eight cases (74%) with intestinal necrosis and twenty cases (26%) without intestinal necrosis. Multivariate analysis of factors associated with IN was organ failure (odds ratio (OR): 4.1; 95% confidence interval (95%CI): 1.26–8.59;* P*=0.028), elevated serum lactate (OR:3.6; 95% CI: 1.51–5.47;* P*=0.024), bowel loop dilation on computerized tomography (CT) scan (OR: 2.8; 95% CI: 1.32–7.23;* P*=0.031), and the time between onset of symptoms and operation (OR: 4.8; 95% CI: 1.36–9.89;* P*=0.012). Area under the receiver operating characteristics curve for the diagnosis of IN with MVT was 0.901 (95%CI: 0.809–0.993; P=0.000) depending on the different number of predictive factors.

**Conclusion:**

Predictive risk factors for IN with MVT were organ failure, elevated serum lactate level, bowel loop dilation on CT, and the time between onset of symptoms and operation. However, this result is from a retrospective study and further long-term, large-sample prospective studies are required to confirm this finding.

## 1. Introduction

Acute mesenteric ischemia (AMI) is a gastrointestinal and vascular emergency that is often underestimated and may be with possibly transmural necrosis of the bowel wall [1]. AMI of venous origin is attributed to mesenteric venous thrombosis (MVT) and acute superior mesenteric venous thrombosis (ASMVT) is a rare but potentially catastrophic clinical complication that may lead to ischemia and/or infarction of the intestine [2, 3]. The diagnosis of ASMVT remains elusive until intestinal gangrene and peritonitis occur [4]. A recent study showed that a multimodal and multidisciplinary management could decrease the rate of intestinal resection, as well as improve short-term and long-term survival [5]. Proper diagnosis at earlier stages became feasible and quick intervention could prevent the progression of bowel ischemia to irreversible necrosis, which can improve prognosis of patients with MVT. The aim of the present study was to identify clinical, laboratory, and radiological features in order to find out the predictive risk factors of IN in patients with MVT, which can discriminate cases with MVT who were in urgent need for surgery intervention.

## 2. Methods

We conducted a retrospective analysis of data pertaining to adult patients presenting with MVT at our hospital (West China hospital, Sichuan University) between January 2014 and May 2018. We reviewed all patients' characteristics, which included gender, age, body mass index (BMI), history of cardiovascular disease, atherosclerosis risk factors (tobacco consumption, alcohol use, and elevated cholesterol or triglycerides), gastrointestinal bleeding, peritonitis (signs of peritonitis (abdominal tenderness, rebound tenderness, and rigidity) and pyocyte or/and bacterial growth were found in ascites), organ failure that included respiratory failure defined as PaO2 / FiO2(mmHg): 76-150; heart failure defined as heart rate (HR) × central venous pressure (CVP) / mean arterial pressure (MAP): 20.1-30); liver failure defined as serum bilirubin (umol/L): 121-240; and kidney failure defined as serum creatinine (umol/L): 351-500, and the time between onset of symptoms and laboratory investigation (complete blood count, liver and kidney function tests, arterial blood gases (ABGs), serum electrolytes, serum lactate level, and DIC test). CT scan features data included bowel wall thickening or thinning, decreased bowel wall enhancement, pneumatosis intestinalis, ascites, portal venous gas, bowel loop dilation, and feces sign (particulate faeculent material mingled with gas bubbles in the lumen of the small intestine, as seen in the colon on CT) and thrombosis of superior mesenteric vein. In our study, we reviewed presence or absence of intestinal necrosis and the clinical treatment including surgical exploration or conservative treatment. This study was approved by the Regional Ethics Committee of our hospital and was performed in accordance with relevant guidelines and regulations. In addition, all patients signed informed consents.

SPSS program was used to analysis the collected data. Continuous data and categorical data were expressed in the form of mean ± standard deviation (SD) and number, percentage, and normal range, respectively. Quantitative variables were analyzed by Student's t test and Chi-square tests were used in the analysis of qualitative variables. When P values of some variables were less than 0.05 in the t test or Chi-square test, a multivariate analysis was used in the analysis of these variables. Multivariate analysis was conducted through binary logistic regression test to determine the independent variables that predicted intestinal necrosis. Results of the multivariate analysis are shown as odds ratio (OR) and 95% confidence interval (95%CI) and we established a model according to the number of risk factors. The accuracy of this model was further evaluated using a receiver operating characteristics curve. All P values were two-sided, and P values of less than 0.05 were considered to indicate statistical significance.

## 3. Results

### 3.1. Patient Characteristics

There were seventy-eight patients (54 males; mean age: 60.9±15.5 (range 24-89) years) with mesenteric vascular thrombosis (MVT) included to the study from January 2014 to May 2018. The mean BMI of patients was 25.9±3.3 (range, 18.4–38.9). Eighteen (23%) patients had more than one associated comorbidity and thirty-four (44%) patients were with cardiac disease (coronary heart disease or atrial fibrillation). Twenty (26%) patients had diabetes mellitus, eighteen (23%) cases had arterial hypertension, and six (8%) cases had chronic liver disease and venous thromboembolism. Regarding clinical signs on admission, there were fifty (64%) patients with peritonitis and thirty-six (46%) cases were with one organ failure. In our study, the mean time between onset of symptoms and operation was 76.6±73.8 (range: 13-360) hours. Baseline characteristics of patients were illustrated in Table 1.

### 3.2. Laboratory Data and Computed Tomography Finding

In our study, the mean white blood cell count was 16.7±6.2 (range: 5.7-32.6) 10^∧^9/L, and the mean platelet count was 194.7±83.7 (range: 80-392) 10^∧^9/L. The mean serum albumin was 36.7±6.8g/L (range: 20.4-47.7g/L) and the mean serum creatinine was 93.8±64.2umol/L (range: 38-372umol/L). The mean arterial pH was 7.3±0.1 (range: 7.1-7.46), the mean serum lactate level was 2.0±0.5mmol/L (range: 1.1-2.8mmol/L), the mean dimer was 10.0±11.1mg/L (range: 0.52-36.82 mg/L), and the mean international normalized ratio (INR) was 1.2±0.2 (range: 0.91-1.97) (Table 1). Abdominopelvic enhanced CT scanning was done in all patients and showed the following findings: eighteen (23%) cases with thinning of bowel wall, forty-eight (62%) patients with thickening of bowel wall, forty-four (56%) patients with dilated bowel loops, seventeen (22%) patients with pneumatosis intestinalis, forty-eight (62%) patients with decreased bowel wall enhancement, sixty-four (82%) patients with ascites, and twenty-four (31%) patients with feces sign (Table 2).

### 3.3. Intestinal Necrosis Assessment

Sixty-two (79%) patients performed laparotomy based on clinical condition, which included abdominal guarding, occurrence of organ failure, and bloody ascites that were shown through diagnostic peritoneocentesis and intraoperative finding. Fifty-eight (74%) patients had evidence of small bowel necrosis intraoperatively and intestinal resection was carried out. Two (3%) patients had reversible bowel ischemia and did not warrant intestinal resection. The other two (3%) patients were with a part of greater omentum necrosis. Sixteen (21%) patients did not undergo surgery, all of whom were regarded as presenting with reversible bowel ischemic injury and recovered without surgical intervention.

### 3.4. Analysis of Risk Factors for Intestinal Necrosis

In our study, t test, Chi-square test, and binary logistic regression test were used to analyze the risk factors for intestinal necrosis. Univariate analysis showed that intestinal necrosis was significantly associated with BMI (P=0.003), peritonitis (P<0.001), organ failure (P=0.009), mean serum lactate levels (P<0.001), arterial PH (P=0.001), and the time between onset of symptoms and operation (P=0.004). The analysis for CT signs revealed that thinning of bowel wall (P=0.004), dilated bowel loops (P<0.001), and decreased bowel wall enhancement (P=0.001) were the significant predictors for intestinal necrosis. The results were illustrated in Tables 1–3.

Multivariate analysis showed that independent risk factors of IN were organ failure (OR: 4.1; 95%CI:1.26–8.59; P=0.028), serum lactate levels (OR: 3.6; 95%CI:1.51–5.47; P=0.024), bowel loop dilation on CT (OR: 2.8; 95%CI:1.32–7.23; P=0.031), and the time between onset of symptoms and operation (OR: 4.8; 95%CI:1.36–9.89; P=0.012) (Table 3). We established a model that included these risk factors of IN, of which bowel loop dilation on CT was more than and equal to 2.0 cm and the value of serum lactate and the time between onset of symptoms and operation was more than and equal to 2.0, 72 hours, respectively. We compared the diagnostic value of one risk factor, two risk factors, and three or more risk factors. The overall area under the receiver operating characteristics curve for the diagnosis of bowel necrosis was 0.901 (95% CI: 0.809–0.993; P=0.000).

## 4. Discussion

AMI is an acute vascular emergency caused by insufficient mesenteric blood flow with subsequent ischemia and possibly results in transmural necrosis of the intestinal wall. AMI of venous origin is attributed to mesenteric venous thrombosis (MVT). ASMVT is an uncommon disease that presents no obvious specific symptoms in the early stage and has an insidious onset of symptom. The mortality rate of acute mesenteric venous thrombosis is usually up to 50% [2, 6, 7]. Thus, preventing the progression from reversible to irreversible intestinal ischemic injury should be a primary goal in the management of AMI [8]. The early diagnosis of intestinal ischemia remains a challenge and further research is required to identify improved serological markers [9].

In our retrospective study, we identified in a multivariate analysis for risk factors of bowel necrosis with MVT. Predictive factors (organ failure, bowel loop dilation on CT, elevated serum lactate, and the time between onset of symptoms and operation) had an additive effect and close monitoring of these factors could help determine whether a surgery was required or not. As previously implied [5], the presence or absence of peritonitis, organ failure, and/or elevated serum lactate levels defined early or late forms of AMI and were significantly associated with the length of stay in the intensive care unit and rate/length of intestinal resection. In line with another study [8] that found that elevated serum lactate levels were significantly associated with irreversible transmural bowel necrosis, we observed that patients with higher serum lactate could develop intestinal necrosis and serum lactate levels were a significant predictor for bowel necrosis in our study. An elevated lactate concentration was considered an important warning signal in patients with abdominal complaints that implies the need of emergent surgery [10].

Clinical presentation of MVT varies greatly and it is difficult to achieve a clinical diagnosis in these patients, leading to a delay in treatment. In our study, the diagnosis of MVT was often obtained at a late stage, which was as a result of patients' delay, or because of doctors' delay in some other hospitals, where they could not undergo immediate surgical treatment. The mean time between onset of symptoms and operation in cases with IN was 90.5±80.3 (13-360) hours; however the time of patients without IN was 36.2±22.5 (15-72) hours. We argue that the more time between onset of symptoms and operation it takes, the more likely it is to result in intestinal necrosis and bowel resection for patients with MVT. Without early and appropriate treatment, the systemic inflammatory caused by ischemic tissue injury and necrotic tissue could cause failure of some organs. The presence of organ failure was significantly associated with intestinal necrosis and resection and acute MVT has high incidence of recurrence (33%–40%) in the early postoperative period, so that many clinicians perform a second-look operation either routinely or selectively [11]. However, no patients performed second-look operation in our study, because they were all unwilling to undergo another operation for a second look.

Earlier identification and resection of intestinal necrosis could reduce morbidity and mortality, as well as improve the functional outcome of the small bowel after MVT. Thirty-day mortality and five-year survival for MVT are strongly associated with “short-bowel” syndrome [12]. Delayed diagnosis in patients with MVT is associated with a high morbidity and mortality. Computed tomography scanning with I.V. contrast is regarded as tool in suspected cases of MVT and multidetector computed tomography (MDCT) is a fundamental imaging technique, which is essential for the early diagnosis of AMI [13–15]. The present study revealed that the onset of intestinal necrosis was associated with these CT signs, of which dilated bowel loops were the radiological sign which was most strongly associated with bowel necrosis. Bowel dilation and decreased mural enhancement combined with pneumatosis intestinalis were highly predictive of nonocclusive mesenteric ischemia. Absence of mural enhancement and the presence of fluid and gas were in late stage venous thrombosis [16, 17]. Thinning of bowel wall on CT was a sign that bowel dilatation and stasis were due to the weakening or disappearance of intestinal peristalsis at the late stage in patients with MVT. Decrease of bowel wall enhancement was found to be a marker of ischemic strangulation in patients with acute bowel obstruction, as well as predictive of mortality in patients presenting with pneumatosis intestinalis [18, 19]. In our study, intestinal necrosis was not significantly or independently associated with pneumatosis intestinalis and portal venous gas on CT, which was the same as the recent study [20, 21].

In line with another study [8], we find that the rate of intestinal necrosis did not significantly increase in the presence of gastrointestinal hemorrhage. Although a previous study on superior mesenteric vein thrombosis found that diabetes mellitus was associated with intestinal resection [22], we cannot reveal that atherosclerosis risk factors and associated comorbidities (diabetes mellitus, cardiac disease, arterial hypertension, chronic liver disease, and venous thromboembolism) were significantly or independently associated with intestinal necrosis in patients with MVT because we reviewed the characteristics of patients with MVT and excluded the cases with mesenteric arterial embolism or thrombosis.

This study has some limitations, such as the relatively small number of cases in our study and being a retrospective study from a single center. Moreover, no data on the clinical outcomes and followup of patients were described. But the aim of the present study was to identify clinical, laboratory, and radiological features and to find out the predictive risk factors of IN in patients with MVT. Our study can provide a possible approach to the issue of intestinal ischemia with MVT.

## 5. Conclusion

Although several studies have clearly identified factors associated with intestinal necrosis in acute mesenteric ischemia, the predictive factors of intestinal necrosis with MVT have yet to be clearly identified. The present study analyzed the baseline characteristics of patients with MVT and concluded that organ failure, bowel loop dilation on CT, elevated serum lactate, and the time between onset of symptoms and operation were the predictive factors for IN with MVT. Close monitoring of these factors should be a part of the management in patients with MVT as it could help avoid unnecessary laparotomy, prevent bowel resection, as well as complications of undiagnosed nonresected necrosis, and possibly lower mortality. However, this result is from a retrospective study, which cannot be seen as a definitive conclusion. Some further long-term, large-sample prospective studies are required to confirm this finding.

## Figures and Tables

**Table 1:** Result of univariate analysis for baseline characteristics of 78 patients with mesenteric vascular thrombosis.

Variable	Intestinal necrosis N=58(%)	NO intestinal necrosis N=20(%)	Overall N=78(%)	*P value*
*Patient demographics *				
Age(years)	60.8±16.4	61.4±13.3	60.9±15.5	0.880
Male/female	38:20	16:4	54:24	0.273
BMI	26.7± 2.6	24.0± 4.5	25.9±3.3	**0.003**
*Atherosclerosis risk factors *				
Tobacco use (%)	42(72)	12(60)	54(69)	0.583
Alcohol use (%)	36(62)	8(40)	44(56)	0.196
Dyslipidemia (%)	28(48)	4(20)	32(41)	0.176
*Associated comorbidities*				
Diabetes mellitus (%)	16(28)	4(20)	20(26)	0.569
Cardiac disease (%)	26(79)	8(40)	34(44)	0.797
Arterial hypertension (%)	14(24)	4(20)	18(23)	0.196
Chronic liver disease %)	4(7)	2(10)	6(8)	0.643
Venous thromboembolism (%)	6(10)	0	6(8)	0.329
More than one comorbidity (%)	16(28)	2(10)	18(23)	0.133
*Clinical signs on admission*				
Mean pulse rate (bpm)	105.7±27.7	101.1±10.9	104.5±24.5	0.474
Mean systolic blood pressure (mmHg)	129.4±24.5	133.8±15.6	130.5±22.4	0.451
Gastrointestinal hemorrhage (%)	12(21)	4(20)	16(21)	0.612
Mean time between onset of symptoms and operation (hours)	90.5±80.3	36.2±22.5	76.6±73.8	**0.004**
Organ failure (%)	32(55)	4(20)	36(46)	**0.009**
Peritonitis (%)	44(76)	6(30)	50(64)	**<0.001**
*Laboratory parameters*				
Mean white blood cell count(10^∧^9/L)	17.1±6.6	15.3±4.7	16.7±6.2	0.253
Mean platelet count (10^∧^9/L)	197.6±81.3	186.3±94.5	194.7±83.7	0.603
Mean serum creatinine (umol/L)	94.9±74.1	90.6±17.7	93.8±64.2	0.797
Mean serum albumin (g/L)	37.1±7.5	35.7±4.1	36.7±6.8	0.436
Mean serum ALT(IU/L)	117.9±517.2	26.7±18.3	109.4±479.7	0.471
Mean serum AST(IU/L)	138.1±555.8	26.3±11.2	94.6±445.9	0.406
Mean serum lactate (mmol/L)	2.1±0.4	1.6±0.3	2.0±0.5	**<0.001**
Mean arterial pH	7.31±0.1	7.39±0.1	7.3±0.1	**0.001**
International normalized ratio (INR)	1.17±0.2	1.15±0.2	1.2±0.2	0.706
Dimer (mg/L)	10.4±11.6	8.9±10.2	10.0±11.1	0.617

BMI: body mass index; ALT: alanine aminotransferase; AST: aspartate aminotransferase.

**Table 2:** Univariate analysis for computed tomography finding of mesenteric vascular thrombosis in 78 patients.

Variable	Intestinal necrosis N=58(%)	NO intestinal necrosis N=20(%)	Overall N=78(%)	*P value *
Thinning of bowel wall (%)	18(31)	0	18(23)	**0.004**
Thickening of bowel wall (%)	34(59)	14(70)	48(62)	0.432
Dilated bowel loops (%)	40(69)	4(20)	44(56)	**<0.001**
Pneumatosis intestinalis (%)	16(28)	1(5)	17(22)	0.056
Decreased bowel wall enhancement (%)	42(72)	6(30)	48(62)	**0.001**
Ascites (%)	50(86)	14(70)	64(82)	0.173
Feces sign (%)	20(34)	4(20)	24(31)	0.273
Portal venous gas (%)	6(1)	0	6(1)	0.308
Combined PV and SMV thrombosis (%)	26(45)	4(20)	30(38)	0.263
Thrombosis of SMV (%)	58(100)	20(100)	78(100)	0.424

PV: portal venous; SMV: superior mesenteric venous.

**Table 3:** Risk factors associated with intestinal necrosis in mesenteric vascular thrombosis on logistic regression analysis.

Variable	*P value *	OR	95%CI
*Patient demographics *			
BMI	0.384	-	-
*Clinical signs on admission*			
Peritonitis	0.246	-	-
Mean time between onset of symptoms and operation	0.012	4.8	1.36-9.89
Organ failure	0.028	4.1	1.26-8.59
*Laboratory parameters*			
Mean serum lactate	0.024	3.6	1.51-5.47
Mean arterial pH	0.088	-	-
*Computed tomography signs*			
Dilated bowel loops	0.031	2.8	1.32-7.23
Thinning of bowel wall	0.328	-	-
Decreased bowel wall enhancement	0.529	-	-

BMI: body mass index; OR: odds ratio; CI: confidence interval.

## Data Availability

No data were used to support this study.

## List of Abbreviations

MVT: Mesenteric venous thrombosis
ASMVT: Acute superior mesenteric venous thrombosis
IN: Intestinal necrosis
CT: Computer scanning
MDCT: Multidetector computed tomography
OR: Odds ratio
CI: Confidence interval
AMI: Acute mesenteric ischemia
BMI: Body mass index
ABGs: Arterial blood gases
SD: Standard deviation
INR: International normalized ratio.

## References

[1] Kärkkäinen J. M., Lehtimäki T. T., Manninen H., Paajanen H. (2015). Acute mesenteric ischemia is a more common cause than expected of acute abdomen in the elderly. *Journal of Gastrointestinal Surgery*.

[2] Acosta S., Ögren M., Sternby N.-H., Bergqvist D., Björck M. (2005). Mesenteric venous thrombosis with transmural intestinal infarction: A population-based study. *Journal of Vascular Surgery*.

[3] Chang R. W., Chang J. B., Longo W. E. (2006). Update in management of mesenteric ischemia. *World Journal of Gastroenterology*.

[4] Glenister K. M., Corke C. F. (2004). Infarcted intestine: A diagnostic void. *ANZ Journal of Surgery*.

[5] Corcos O., Castier Y., Sibert A. (2013). Effects of a multimodal management strategy for acute mesenteric ischemia on survival and intestinal failure. *Clinical Gastroenterology and Hepatology*.

[6] Acosta S. (2015). Mesenteric ischemia. *Current Opinion in Critical Care*.

[7] Abdu R. A., Zakhour B. J., Dallis D. J. (1987). Mesenteric venous thrombosis: 1911 to 1984. *Surgery*.

[8] Nuzzo A., Maggiori L., Ronot M. (2017). Predictive factors of intestinal necrosis in acute mesenteric ischemia: prospective study from an intestinal stroke center. *American Journal of Gastroenterology*.

[9] Evennett N. J., Petrov M. S., Mittal A., Windsor J. A. (2009). Systematic review and pooled estimates for the diagnostic accuracy of serological markers for intestinal ischemia. *World Journal of Surgery*.

[10] Lange H., Toivola A. (1997). Warning signals in acute abdominal disorders. Lactate is the best marker of mesenteric ischemia. *Läkartidningen *.

[11] Paterno F., Longo W. E. (2008). The etiology and pathogenesis of vascular disorders of the intestine. *Radiologic Clinics of North America*.

[12] Abu-Daff S., Abu-Daff N., Al-Shahed M. (2009). Mesenteric venous thrombosis and factors associated with mortality: A statistical analysis with five-year follow-up. *Journal of Gastrointestinal Surgery*.

[13] Yikilmaz A., Karahan O. I., Senol S., Tuna I. S., Akyildiz H. Y. (2011). Value of multislice computed tomography in the diagnosis of acute mesenteric ischemia. *European Journal of Radiology*.

[14] Moschetta M., Telegrafo M., Rella L. (2014). Multi-detector CT features of acute intestinal ischemia and their prognostic correlations. *World Journal of Radiology*.

[15] Bala M., Kashuk J., Moore E. E. (2017). Acute mesenteric ischemia: Guidelines of the World Society of Emergency Surgery. *World Journal of Emergency Surgery*.

[16] Barrett T., Upponi S., Benaglia T., Tasker A. D. (2013). Multidetector ct findings in patients with mesenteric ischaemia following cardiopulmonary bypass surgery. *British Journal of Radiology*.

[17] Reginelli A., Genovese E. A., Cappabianca S. (2013). Intestinal ischemia: US-CT findings correlations. *Critical Ultrasound Journal*.

[18] Millet I., Taourel P., Ruyer A., Molinari N. (2015). Value of CT findings to predict surgical ischemia in small bowel obstruction: A systematic review and meta-analysis. *European Radiology*.

[19] Lee H.-S., Cho Y.-W., Kim K.-J., Lee J. S., Lee S. S., Yang S.-K. (2014). A simple score for predicting mortality in patients with pneumatosis intestinalis. *European Journal of Radiology*.

[20] Furuya Y., Yasuhara H., Ariki K. (2002). Hepatic portal venous gas caused by blunt abdominal trauma: Is it a true ominous sign of bowel necrosis?: Report of a case. *Surgery Today*.

[21] Tilsed J. V. T., Casamassima A., Kurihara H. (2016). ESTES guidelines: acute mesenteric ischaemia. *European Journal of Trauma and Emergency Surgery*.

[22] Elkrief L., Corcos O., Bruno O. (2014). Type 2 diabetes mellitus as a risk factor for intestinal resection in patients with superior mesenteric vein thrombosis. *Liver International*.

